# Single‐gene speciation: Mating and gene flow between mirror‐image snails

**DOI:** 10.1002/evl3.31

**Published:** 2017-11-21

**Authors:** Paul M. Richards, Yuta Morii, Kazuki Kimura, Takahiro Hirano, Satoshi Chiba, Angus Davison

**Affiliations:** ^1^ School of Life Sciences University of Nottingham Nottingham NG7 2RD United Kingdom; ^2^ Division of Ecology and Evolutionary Biology, Graduate School of Life Sciences Tohoku University Aobayama Sendai 980–8578 Japan

**Keywords:** Behavioral genetics, evolutionary genomics

## Abstract

Variation in the shell coiling, or chirality, of land snails provides an opportunity to investigate the potential for “single‐gene” speciation, because mating between individuals of opposite chirality is believed not possible if the snails mate in a face‐to‐face position. However, the evidence in support of single‐gene speciation is sparse, mostly based upon single‐gene mitochondrial studies and patterns of chiral variation between species. Previously, we used a theoretical model to show that as the chiral phenotype of offspring is determined by the maternal genotype, occasional chiral reversals may take place and enable gene flow between mirror image morphs, preventing speciation. Here, we show empirically that there is recent or ongoing gene flow between the different chiral types of Japanese *Euhadra* species. We also report evidence of mating between mirror‐image morphs, directly showing the potential for gene flow. Thus, theoretical models are suggestive of gene flow between oppositely coiled snails, and our empirical study shows that they can mate and that there is gene flow in *Euhadra*. More than a single gene is required before chiral variation in shell coiling can be considered to have created a new species.

**Impact Summary**

Although most snails have a right‐handed spiraling shell, rare “mirror‐image” individuals have a shell that coils to the left. This curious inherited condition has attracted attention because the genitals of mirror image snails are on different sides of the head, and so mating is difficult or impossible. If they are unable to mate, then does a change in the direction of the shell coil make a new species? In investigating a Japanese snail genus, *Euhadra*, we were surprised to find that different‐coiling individuals can sometimes mate, against expectations, and that there is evidence for this in their genetic make‐up. It turns out that the mating problem is mainly behavioral, rather than a physical incompatibility. This new work therefore suggests that the two types of Japanese snail should be considered a single species, and has implications for the classification of other snail species. As it is has previously been shown that the same sets of genes that make mirror image snails are also involved in making mirror image bodies in other animals–including humans–then further research using the natural variation snails could offer the chance to develop an understanding of how organs are placed in the body and why this process can sometimes go wrong.

Understanding the extent and underlying causes of speciation under gene flow is a longstanding challenge in evolutionary biology. Strong reproductive isolation usually depends upon the evolution and maintenance of associations between multiple traits contributing to different reproductive barriers (Coyne and Orr 2004). However, a problem is that gene flow is fundamentally antagonistic to this process because it is expected to homogenize divergence at individual loci, and through recombination, randomize associations between the different loci contributing to reproductive isolation (Felsenstein 1981; Coyne and Orr 2004; Gavrilets 2004; Servedio 2009). Consequently, the complete cessation of gene flow by means of geographic isolation has traditionally been viewed as necessary for reproductive isolation to evolve. Unopposed by recombination, processes such as mutation, selection, and genetic drift can drive genome‐wide divergence between allopatric populations, leading to the build‐up of linkage disequilibrium between loci contributing to reproductive barriers (Felsenstein 1981; Coyne and Orr 2004).

Despite the theoretical difficulties, it is now clear that speciation with gene flow may be relatively common in nature (Servedio and Noor 2003; Gavrilets 2004; Bolnick and Fitzpatrick 2007; Nosil 2008; Smadja and Butlin 2011). For instance, *de novo* divergence in sympatry may occur through assortative mating resulting from associations between loci subject to divergent ecological selection (e.g., differential local adaptation to habitat, or predation) and loci underlying mating traits (Rundle and Nosil 2005). Alternatively, geographic isolation may be important for initiating speciation, with divergent ecological selection, or reinforcement strengthening reproductive barriers following secondary contact (Servedio and Noor 2003; Rundle and Nosil 2005). The challenge is in determining the relative contributions of spatial isolation and gene flow to the evolution of reproductive isolation (Smadja and Butlin 2011; Martin et al. 2013), and elucidating mechanisms that act in lieu of spatial isolation to prevent recombination from disrupting associations between the different components of reproductive isolation (Smadja and Butlin 2011).

One exceptional means by which speciation with gene flow could be facilitated is through occasional reversals of left‐right asymmetry, or *chirality*, in snails. Due to pleiotropic effects of the maternal effect locus that determines snail chirality (Boycott and Diver 1923; Sturtevant 1923; Schilthuizen and Davison 2005), mating is believed not possible between mirror‐image individuals with low spired shells. Switches in chirality may therefore be a driver of so‐called “single‐gene” speciation (Gittenberger 1988; Asami et al. 1998; Coyne and Orr 2004; Schilthuizen and Davison 2005; Hoso et al. 2010). However, the likelihood of single‐gene speciation in snails, and the mechanisms by which it could occur have been the subject of much debate because it is both theoretically challenging (Johnson et al. 1990; Orr 1991; Davison et al. 2005) and the empirical evidence is extremely limited.

First, theoretical models have shown that while individual snails of opposite coil may be unable to mate, gene flow could be substantial between morphs. As the chiral phenotype of offspring is determined by the maternal genotype, occasional chiral reversals will take place and enable gene flow, unless there is complete reciprocal fixation of chirality‐determining alleles (Davison et al. 2005).

Second, as predicted by classic two‐locus models of speciation (Orr 1996), fixation of a novel chiral allele is unlikely because the new chiral morph might lack potential intrachiral mating partners (Johnson 1982; Orr 1991, 1996). Consequently, several studies have investigated the conditions under which this mating disadvantage could be overcome, including founder effects, population size, and density, as well as selection, such as reproduction character displacement or predation (Johnson 1982; Orr 1991; van Batenburg and Gittenberger 1996; Davison et al. 2005; Yamamichi and Sasaki 2013).

Third, sparse empirical data mean that putative instances of single‐gene speciation have been inferred from single‐gene mitochondrial phylogenies (Ueshima and Asami 2003; Davison et al. 2005; Uit de Weerd et al. 2006; Feher et al. 2013; Modica et al. 2016), or by combining single mitochondrial genes with relatively invariable ribosomal RNA sequences (Hoso et al. 2010; Kornilios et al. 2015). From this data alone, it is impossible to definitively distinguish between low levels of gene flow, introgressive hybridization, or speciation.

Finally, the other main approach has been to investigate and compare patterns of chiral variation between species, across wide geographical scales (Hoso et al. 2010; Gittenberger et al. 2012). While this is useful in understanding broad patterns, especially in explaining the high frequency of sinistrals in South East Asia (Hoso et al. 2010), the phylogenetic relationship between the species is often not clear, and beset by the taxonomic problem that species are sometimes defined on the basis of chirality alone.

Chiral reversal in the Japanese snail genus *Euhadra* perhaps presents one of the best candidate systems for investigating the potential for single‐gene speciation, but also illustrates the lack of empirical data. Two independent studies (Ueshima and Asami 2003; Davison et al. 2005) have used mitochondrial DNA sequences to investigate the phylogenetic relationships between the five sinistral *Euhadra* species and the other dextral species. Both phylogenies supported a single origin of the sinistral species from a dextral ancestor, but also found evidence supporting recent evolution of dextral *E. aomoriensis* from sinistral *E. quaesita*. Specifically, three lineages of the dextral species were polyphyletically distributed within *E. quaesita*, leading to the suggestion that this is due repeated single‐gene speciation of the dextral from the sinistral (Ueshima and Asami 2003). We therefore set out to test the evidence for single‐gene speciation in *Euhadra*, by combining a fine‐scale RAD‐seq phylogeographic study with behavioral observations of snail mating.

## Methods

### SAMPLING

There are 22 taxonomically defined species/subspecies of *Euhadra* (Bradybaenidae) distributed throughout Japan and the neighboring Korean island of Jeju (Davison et al. 2005). For this study, three of the five sinistral species, *E. decorata*, *E. murayamai*, and *E. quaesita*, the dextral species *E. senckenbergiana*, and the nominal dextral species *E. “aomoriensis*” were sampled.

**Figure 1:**
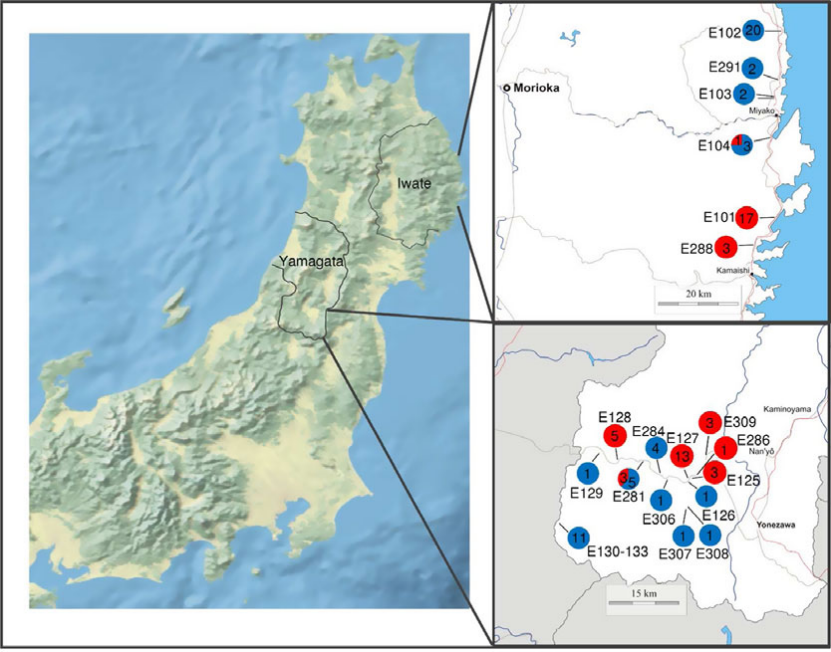
Map showing topography of northern and central Honshu, Japan. Insets: two newly identified contact zones between sinistral (red) *E. quaesita* and dextral (blue) *E. aomoriensis*, showing sample size and site ID.

Sinistral *E. quaesita* were collected from across the Tohoku (northern Honshu) region of Japan. *E. aomoriensis* has a distribution that is largely allopatric with *E. quaesita*, being more frequently found in sympatry with sinistral *E. decorata*, especially in the northern part of Tohoku. Like *E. quaesita*, *E. aomoriensis* was also sampled opportunistically across Tohoku, but with a concentrated effort on two dextral/sinistral contact zones that we identified, one in Iwate prefecture (NE Tohoku) and another in Yamagata (SW Tohoku), approximately 250 km apart (Fig. 1). Further samples were obtained of sinistral *E. murayamai*, a species that is endemic to a small limestone outcrop and is also polyphyletic within *E. quaesita*, based on mtDNA (Davison et al. 2005). Finally, *E. senkenbergiana* and *E. decorata* were included because they are sometimes sympatric with dextral *E. aomoriensis* and sinistral *E. quaesita*. Thus, the collection contained samples of sympatric and parapatric *E. quaesita/E. aomoriensis*, and for comparison, sympatric *E. quaesita*/*E. senckenbergiana*, and *E. aomoriensis*/*E. decorata*.

### BEHAVIORAL OBSERVATIONS

It is commonly assumed that dextral and sinistral low‐spired snails are either unable to mate, or can only mate very rarely (Asami et al. 1998; Davison and Mordan 2007). There are no known reports of mating, and we are not aware of any systematic studies. We used a network of malacological contacts, and a knowledge of Japanese language sources to investigate evidence of possible matings between dextral and sinistral *Euhadra*.

### DE NOVO GENERATION OF RAD‐SEQ SNP MARKERS

RAD‐seq was used to generate SNP markers for 16 individuals representing four species. The samples included two sinistral *E. quaesita* populations (*n* = 6) that are largely parapatric with two dextral *E. aomoriensis* populations (*n* = 6) in East Iwate and South Yamagata, where geographic and mtDNA data suggest interchiral contact may have been recent or ongoing (see Results). For comparison, one population of dextral *E. senkenbergiana* (*n* = 3) and one individual of sinistral *E. decorata* were used.

From the final filtered set of SNPs (see Supplementary Methods for further details) a number of datasets were generated, allowing for varying degrees of missing data. After quality filtering, 13,167 biallelic loci were found in eight or more individuals, which reduced to 7871 loci once singleton SNPs were removed. There were still a substantial number of missing genotypes in this dataset, so to refine the loci used further, only one null was allowed in each of the four main population samples of interest (sinistral and dextral snails from Iwate and Yamagata), leaving 4598 loci. This reduced dataset was used for all subsequent analyses. Although not shown, other datasets produced similar outputs in terms of subsequent analyses.

### RAD‐SEQ PHYLOGENOMIC ANALYSES

We conducted four separate phylogenomic analyses, based on the RAD‐seq dataset. First, the concatenated SNPs were used to build a maximum likelihood phylogeny, using the same methods as for the mitochondrial data. However, phylogenies are not useful in understanding conflicting signals in the underlying data, as might be produced by varying degrees of linkage between markers, recombination, and introgression. Therefore, we also constructed a network, using the neighbor‐net method in SplitsTree 4 (Huson and Bryant 2006), based on a matrix of uncorrected p‐distances, and using the equal‐angle split transformation and ignoring ambiguous states. Also, the relationship between the individuals was investigated using principal components analysis (PCA), conducted using ADEGENET (Jombart 2008), and ADE4 (Dray and Dufour 2007) in R 3.2.3.

To test for signals of admixture between population samples, and correspondingly, whether any inferred tree is truly bifurcating, we used Treemix (Pickrell and Pritchard 2012). This software uses allele frequencies within groups to relate a sample of populations to their common ancestor, including as output a maximum‐likelihood (ML) tree of estimated migration events, including the direction. Five populations used were Yamagata sinistral, Yamagata dextral, Iwate sinistral, Iwate dextral, and *E. senckenbergiana* outgroup. F*_ST_* between sites was also estimated, using Genepop and the same populations (Rousset 2008). To complement these analyses, population structure and admixture was estimated using individual genotypes with STRUCTURE v2.3.4 (Falush et al. 2003; Evanno et al. 2005).

### TESTING OF SCENARIOS VIA APPROXIMATE BAYESIAN COMPUTATION METHOD

We used an approximate Bayesian computation (ABC) approach, implemented in the software DIYABC v 2.1.0 (Cornuet et al. 2014) to compare hypotheses. In brief, simulated datasets were produced for five scenarios, by sampling parameter values in defined prior distributions. Three scenarios were similar in that the populations showed a bifurcating topology, only differing in divergence order. Two other models included ancestral admixture, because the shared chirality between dextral *E. aomoriensis* from Yamagata and Iwate might be because of shared ancestry. The analysis was restricted to the four population samples of *E. quaesita* and *E. aomoriensis*, primarily because the large genetic distance between *E. senckenbergiana* and the other samples meant that it was difficult to find a suitable range of parameter values. See Supplementary Methods for further detail.

### MITOCHONDRIAL PHYLOGENETIC AND POPULATION ANALYSES

The number of individuals in the phylogenomic analysis was necessarily limited by resources.1 To sample more individuals and over a greater geographic area, ∼800 bp fragments of 16S rRNA were amplified and sequenced using standard conditions and buffers (see Supplementary Methods). For maximum likelihood phylogenies, an appropriate model of evolution was selected using jModelTest and the Akaike Information Criterion (Darriba et al. 2012), followed by tree construction and visualization using PhyML (Guindon and Gascuel 2003) and TreeExplorer, including bootstrap support, with the tree rooted on *E. senkenbergiana* (Ueshima and Asami 2003; Davison et al. 2005).

## Results

### CONTACT ZONES BETWEEN SINISTRAL AND DEXTRAL SNAILS

In both East Iwate and in South Yamagata prefecture we found one site (E104 and E281, respectively) that contained both chiral morphs of the two species (Fig. 1). Sympatric sites were also recorded between sinistral *E. quaesita* and dextral *E. senckenbergiana* in Chubu (e.g., Noto peninsula, site E261, Anamizu), between sinistral *E. quaesita* and dextral *E. senckenbergiana* in Chubu (D24, Mt. Myojo), and between dextral *E. aomoriensis* and sinistral *E. decorata* in Tohoku (E106, Kabayama; E227, Nohira; E299, Wakasennin; D31, Tamayama).

### MATING BETWEEN DEXTRAL AND SINISTRAL SNAILS

**Figure 2:**
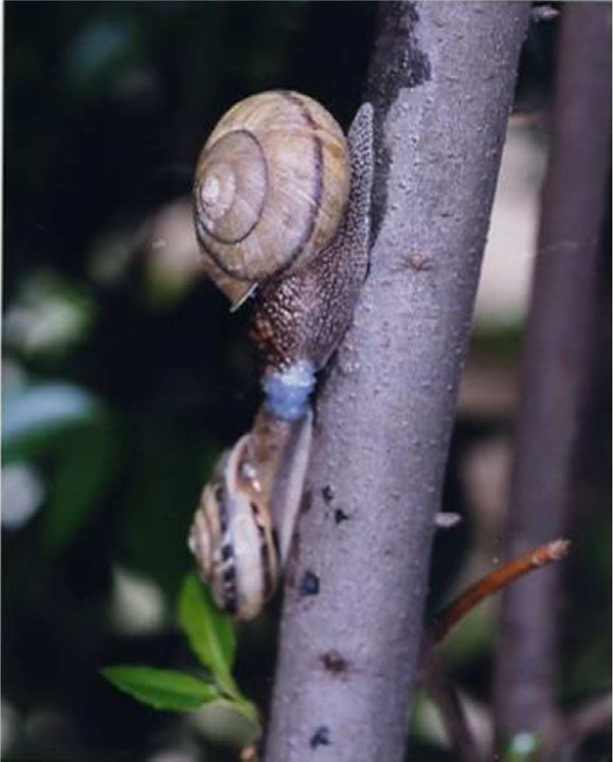
Reciprocal mating between sinistral *E. quaesita* and dextral *E. peliomphala*. Mating between these distantly related species may not produce viable offspring, but illustrates the general point that dextrals and sinistrals are able to mate, if only rarely. Photo: Kentaro Nakao and Seiichi Takase, reproduced with permission.

We found five records of mating between dextral and sinistral *Euhadra* (Table 1; Fig. 2). These observations included matings between sympatric sinistral *E. quaesita* and dextral *E. senckenbergiana*.

**Table 1:** Reports of mating between dextral and sinistral *Euhadra* species

Dextral		Sinistral			Year	Observer	Notes	
*E. senckenbergiana*	Nada, Osaka	*E. quaesita*	Nada, Osaka	Wild	1980	Hiroyuki Nishitani	Pers. comm.	Reciprocal dart shooting observed
*E. senckenbergiana*		*E. quaesita*		Wild	1979	Kazuhisa Shinagawa	Lecture, Hanshin Shell Club	
*E. peliomphala*	Setagaya, Tokyo	*E. quaesita*	Setagaya, Tokyo	Wild	1999	Seiichi Takase, Kentaro Nakano	Report^1^	see Figure 1
*E. amaliae*	Kobe	*E. grata*	Katsuki, Niigata	Laboratory	1982	Ryoji Takada	Pers. comm.	Prolonged mating
*E. sandai*	Mita, Hyogo	*E. quaesita*	Miyakejima	Laboratory	1981	Ryoji Takada	Pers. comm.	Mating on branch in aquarium

^1^Heterospecific mating between sinistral and dextral species in *Euhadra*. Kainakama 33(4): 1, Hanshin Molluscan Research Group, Nishinomiya.

### PHYLOGENOMICS

**Figure 3:**
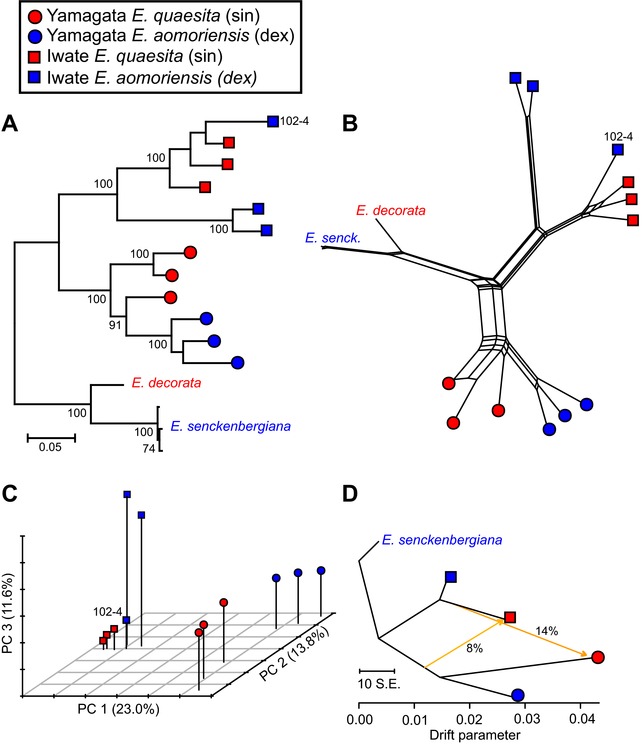
Phylogenomic analysis of 4598 RAD‐seq derived biallelic loci. (A) Maximum likelihood phylogeny using the GTR model, with bootstrap support. (B) Neighbor‐net broadly shows the same relationship between individuals. (C) Principal components analysis, carried out on only *E. quaesita* and *E. aomoriensis*, separates individuals by region (Iwate or Yamagata), then by sites within regions. (D) Treemix analysis of allele frequencies within populations indicates evidence of ancestral migration between populations.

Phylogenies (Fig. 3A–B) based on whole genome RAD‐seq data (see Table S1 for read depths) clearly showed that dextral *E. aomoriensis* and sinistral *E. quaesita* group together and are distinct from sinistral *E. decorata* and dextral *E. senkenbergiana*. Within the *E. quaesita*/*E. aomoriensis* groups, dextrals and sinistrals grouped together by geographic region, Yamagata or Iwate, rather than by chirality, with strong bootstrap support. Within regions, individuals clustered with other individuals from the same sampling location, with the exception of dextral individual E102‐4, which clustered with sinistral individuals from the nearby site, E101. This general result was confirmed using a principal components analysis (Fig. 3C). In the latter, when the analysis was restricted to just *E. quaesita* and *E. aomoriensis* samples, the first three axes explained 48.4% of the variation, respectively separating individuals by region (Iwate or Yamagata, 23.0%), then dextral and sinistrals within Yamagata (13.8%) and dextral and sinistrals within Iwate (11.6%). As above, the position of dextral individual E102‐4 was different to the other two individuals from the same site.

Using population allele frequencies, a TreeMix phylogeny (Fig. 3D) showed the same overall topology, except also containing two putative migration events, from sinistral *E. quaesita* in Iwate into sinistral *E. quaesita* in Yamagata (14%) and from the dextral or sinistral ancestor of the Yamagata snails into sinistral Iwate *E. quaesita* (8%). Using individual genotypes, STRUCTURE identified evidence for more recent gene flow. When the analysis was confined to dextral *E. aomoriensis* and sinistral *E. quaesita* from Iwate, the “optimal” K was 2, but with the dextral E102‐4 clustering with the other sinistrals. When dextral *E. aomoriensis* and sinistral *E. quaesita* from Yamagata were analyzed together, the optimal number of clusters was three, but with sinistrals and dextrals showing mixed ancestry. Estimates of F_ST_ (Table S2) showed that divergence within regions is low to moderate (e.g., F_ST_ ∼0.2 between sinistral and dextral sites in Yamagata), higher between different regions (e.g., Yamagata – Iwate, F_ST_ ∼0.4) and very high when comparing with *E. senckenbergiana* (F_ST_ ∼0.6).

### TESTING OF SCENARIOS

**Figure 4:**
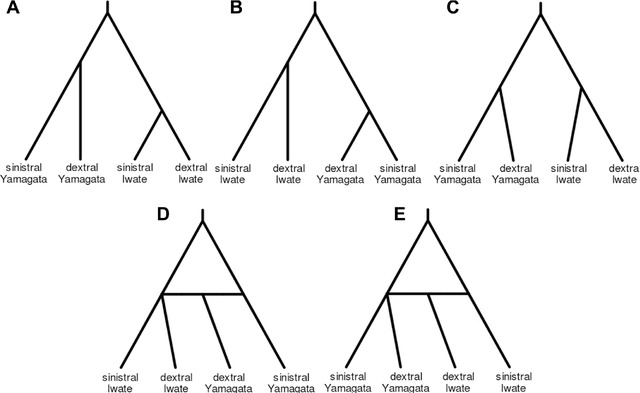
The five scenarios tested in the ABC analysis, three with bifurcating topologies (A, B, C), which only differ in the relative timing of events, and two with admixture between dextral populations (D, E).

Approximate Bayesian computation (Cornuet et al. 2014) was used to compare five different models of cladogenesis, two including admixture (Fig. 4). A scenario involving admixture (0–10%) from dextral Yamagata snails into Iwate snails was the optimal model (model #4 in Table 2; see also Fig. 4), significantly better using logistic regression. To further evaluate confidence in the models, test datasets were simulated. The true scenario had the highest posterior probability for 0.72/0.73 (direct/logistic) test datasets, giving a posterior error rate of 0.27/0.28 (Table 2). Similarly, test datasets were simulated and a prior based error analysis conducted to understand the probability with which true models might be rejected. The proportion of wrongly identified scenarios was 0.33/0.25 (direct/logistic). Finally, scenario specific prior error rate was estimated, by drawing test datasets from the parameter prior distribution under a given scenario. By drawing pods against model #4, and comparing to the next best model (#1), the type I error was 0.10/0.09; by drawing pods against model #1, and comparing to model #4, the type II error for model #1 was 0.29/013. In both cases, similar values were obtained when comparing against other models.

**Table 2:** Scenarios for the repeated evolution of dextral populations of snails within a sinistral species, either independently (1–3), or involving admixture

		**Direct**		**Logistic regression**	
		Posterior	95%	Posterior	95%
	**Scenarios**	probability	confidence	probability	confidence
	**Independent origin** of dextral/sinistrals in Yamagata versus Iwate				
1.	Yamagata snails diverged first	0.27	[0–0.6]	0.03	[0.03]
2.	Iwate snails diverged first	0.12	[0–0.41]	0	[0]
3.	Diverged at same time	0.26	[0–0.65]	0	[0]
	**Shared ancestry** between dextrals (admixture)				
**4**.	**Admixture (<10%) from dextral Yamagata snails into Iwate snails**	**0.37**	**[0–0.79]**	**0.97**	**[0.96–0.97]**
5.	Admixture (<10%) from dextral Iwate snails into Yamagata snails	0	[0]	0	[0]

The best model is highlighted in bold.

### MITOCHONDRIAL PHYLOGENIES

In the Iwate contact zone, it was found that most sinistral and dextral *E. quaesita* and *E. amoriensis* snails were of the same haplogroup, QUA2a, including three haplotypes were shared between sinistral and dextral coiling snails; in the Yamagata contact zone, the same two species were mostly of haplogroup QUA1 (Fig. S1). In comparison, in sites containing *E. quaesita*/*E. senckenbergiana* or *E. aomoriensis*/*E. decorata*, different species contained divergent mitochondrial haplogroups (Figs. S1 and S2; Table S3).

## Discussion

Previously, we used a theoretical model to show that occasional chiral reversals may take place and enable gene flow between mirror image snail‐shell morphs. This is because the maternal inheritance of snail chirality means that there is sometimes a discord between phenotype and genotype, leading to gene flow between different types (Davison et al. 2005). By collecting together reports and observations from Japanese naturalists, we found direct evidence that dextral and sinistral *Euhadra* are sometimes able to mate. Moreover, the genomic data indirectly suggests recent or ongoing gene flow between sinistral *E. quaesita* and dextral *E. aomoriensis*. Thus, overall, the theoretical model (Davison et al. 2005), behavioral observations, genomic data, and biogeographic context do not support a model of single‐gene speciation. More than a single gene is required before chiral variation can be considered to have created a new species, at least in *Euhadra*.

A fundamental new insight from this work is that analyses of genome‐wide SNP markers show that dextral and sinistral *Euhadra* in Iwate, Japan are distinct from dextral *Euhadra* in Yamagata. Moreover, genomic and mtDNA divergence was low in both locations where dextral and sinistral morphs were found together (Figs. 3, S1; Table S3), especially in comparison to other snails that show greater differentiation over shorter geographic distances (Davison and Clarke 2000). Some dextral and sinistral snails shared identical mtDNA haplotypes, and individual snails showed traces of mixed ancestry (Fig. 3A–C; also STRUCTURE analyses). There are only two explanations for this pattern–‐either there is ongoing gene flow between dextral *E. aomoriensis* and sinistral *E. quaesita* in two separate locations, or else there was gene flow, but this has recently ceased.

A second important insight from this work is that face‐to‐face mating between low‐spired snails sometimes take place, albeit at an unknown frequency. Previously, the assumption has been that the genitals are on the “wrong” side of the head in mating between sinistral and dextral snails, and so intromission is not possible. However, the data here suggest that the problem is mainly behavioral. As in *Amphidromus*, the only snail genus that routinely has interchiral mating (Schilthuizen et al. 2007; Schilthuizen and Looijestijn 2009), it is likely that the long, thin, flexible genital organs are able to twist to match the partners chirality. Moreover, in high‐spired snails, it is already known that interchiral mating (by “shell‐mounting”) is less of an issue–‐and AFLP markers have recently been used to show that gene flow is extensive between the two types, as expected (Koch et al. 2017).

Putting our findings together, we are able to reinterpret previous studies (Ueshima and Asami 2003; Davison et al. 2005). The fact that both the mtDNA and nuclear trees presented here are concordant for the polyphyletic pattern puts beyond doubt the hypothesis that dextral *E. aomoriensis* and sinistral *E. quaesita* are coderived, and should probably be treated as forms of the same species, *E. quaesita*. Although it is possible—or even likely—that the dextral chirality determining allele is ultimately derived from another dextral species, *E. aomoriensis* must have mainly shared ancestry with *E. quaesita*, because otherwise we would have instead expected to observe a signal of admixture with dextral *E. senckenbergiana* in the genomic RAD‐seq data. The two chiral types cannot be defined as separate species, given that they are sometimes found in sympatry, they sometimes mate, the genomic evidence for gene flow between the two types, and the underlying theory that reproductive isolation is unstable (Davison et al. 2005). Altogether, these observations critically weaken the argument for chirality directly leading to single‐gene speciation, at least without implicating other factors such as ecology or predation.

A challenging question to consider how dextral *E. aomoriensis* evolved from sinistral *E. quaesita*, or indeed, whether sinistrals evolved from dextrals? Do the geographically separate regions represent independent transition events from sinistral to dextral, or did dextrality evolve once, then introgressing with a local sinistral in secondary contact? Although further investigations are required and the precise details differ, both the Treemix analysis and the ABC results are consistent with past admixture, suggesting that a common origin is likely. Specifically, the ABC analysis is suggestive of admixture from dextral Yamagata snails into Iwate (Table 1), and thus, that the dextrals in both locations may share the same dextral‐determining allele. Alternatively, the Treemix analysis (Fig. 3D) suggests admixture in the opposite direction, as well as from an ancestor of unknown chirality.

This gene flow need not have been direct. As has been suggested in parallel incipient speciation by local adaptation in other species like intertidal *Littorina* snails (Butlin et al. 2008) and sticklebacks (Colosimo et al. 2005), a feasible scenario is that chirality‐determining alleles exists at low frequencies, especially the recessive version, but under certain selective and/or chance biogeographic conditions (see below) reach fixation, establishing a new chiral morph (Johnson 1982; Orr 1991; van Batenburg and Gittenberger 1996; Davison et al. 2005; Hoso et al. 2010; Yamamichi and Sasaki 2013). Unfortunately, it is unknown which allele is dominant in *Euhadra* and this may vary, depending upon genomic context (Clarke and Murray 1969; Schilthuizen and Davison 2005).

A similar interpretation may also be applied to a recent AFLP study on *Alopia* door snails (Koch et al. 2017) and another mitochondrial rRNA study between sinistral and dextral *Satsuma* species (Hoso et al. 2010), a genus that is relatively closely related to *Euhadra*, and which might even share the same chiral‐varying alleles. In the latter, the authors concluded that the presence of multiple sinistral lineages cannot be explained by introgression via hybridization, because *Satsuma* snails mate face‐to‐face. Our data instead suggest that both gene flow via maternal inheritance and direct face‐to‐face mating should be considered in interpreting whether the patterns are really caused by a “speciation gene.”

Evidently, further work is needed to disentangle the geographic context of the chiral reversion event (s) that lead to the evolution of new chiral types in snails. Such a challenge is fundamental to the field of speciation in general (Coyne and Orr 2004; Smadja and Butlin 2011; Martin et al. 2013). More broadly, part of the interest in snail chirality arises from attempts to understand chiral invariance across the metazoans (Grande and Patel 2008; Okumura et al. 2008; Davison et al. 2009; Utsuno et al. 2011; Davison et al. 2016). Thus, while chiral variation in snails is perhaps a small step toward speciation, this chiral variation may be an invaluable genetic resource in helping reveal the earliest steps of symmetry breaking across the Bilateria (Davison et al. 2016).

Associate Editor: Z. Gompert

## Supporting information


**Figure S1**. Phylogenetic relationships between *Euhadra* mitochondrial 16S rRNA haplotypes, rooted on sinistral *E. decorata* (pale grey) and dextral *E. senckenbergiana* (dark grey).
**Figure S2**. Geographic distribution of sampled sinistral and dextral *Euhadra* populations and their corresponding 16S rRNA haplogroups across northern and central Honshu, Japan.

**Table S1**. Post quality‐filtering read counts for the 16 individuals that were included in the RAD‐Seq study.

**Table S2**. Estimates of F*_ST_* between population samples, based on 4898 RAD‐seq‐derived SNPs. F*_ST_* value between parapatric sinistrals and dextrals are shown in bold.

**Table S3**. Location and statistics for the samples used in the mitochondrial samples.

**Table S4**. The 13167 biallelic RAD‐seq loci that were found in eight or more individuals.

**Table S5**. Alignment of mtDNA haplotypes and associated Genbank accession numbers.
