# Supplementary material for: Single‐gene speciation: Mating and gene flow between mirror‐image snails
Source: Evol Lett. 2017 Nov 21;1(6):282–91. doi: 10.1002/evl3.31 (PMC6121799; doi:10.1002/evl3.31)
Supplement: Supplementary file 1 — Figure S1. Phylogenetic relationships between Euhadra mitochondrial 16S rRNA haplotypes, rooted on sinistral E. decorata (pale grey) and dextral E. senckenbergiana (dark grey). Figure S2. Geographic distribution of sampled sinistral and dextral Euhadra populations and their corresponding 16S rRNA haplogroups across northern and central Honshu, Japan. [file EVL3-1-282-s001.docx]

**Supplementary Methods**

***De novo* generation of RAD-seq SNP markers.** RAD-seq was used to generate SNP markers for 16 individuals, representing four species. The samples included two sinistral *E. quaesita* populations (n=6) that are largely parapatric with two dextral *E. aomoriensis* populations (n=6) in East Iwate and South Yamagata, where geographic and mtDNA data suggest interchiral contact may have been recent or ongoing (see results). For comparison, one population of dextral *E. senkenbergiana* (n=3) and one individual of sinistral *E. decorata* were used.

The 16 individuals were initially prepared as two separate libraries, then proportionally pooled together into a single library to achieve more uniform coverage across individuals ([Baxter *et al.* 2011](#_ENREF_1)). The library was sequenced on a single lane of an Illumina HiSeq 2000 machine at the GenePool Genomics Facility, University of Edinburgh, producing 51 bp single-end reads. Overall sequencing quality of the raw reads was assessed using FASTQC 0.10. Average phred quality scores were high across all 51 bases, with at least 75% of reads at any base having a phred score ≥ 30. Some degradation in quality (elevated numbers of sequences with phred scores < 30) was observed towards the end of the read and also in the first base of the barcode; this information was used to inform the read cleaning parameters.

The raw reads were cleaned and processed into putative RAD loci using STACKS 1.8 ([Catchen *et al.* 2013](#_ENREF_2)). Reads were trimmed to 45 bp and then quality checked using a 10% sliding window; if the average phred score dropped below 20 in any window the read was discarded. Reads were also discarded if the barcode region, or *Sbf*I restriction-site contained errors, or if adapter sequence was detected in the read. In the case of three samples it was clear that a substantial number of reads had been discarded due to a single error in the first base of the barcode; therefore these sequences were recovered.

For each individual, USTACKS was used to cluster identical reads together into unique ‘stacks’ of five or more reads. Excessively large stacks were discarded as likely sequencing error, or repetitive loci. Stacks were then clustered using a mismatch threshold of one base to account for variation between alleles. Secondary reads containing sequencing errors, or those which were supported by less than three identical reads were then aligned to these putative loci to increase coverage for SNP calling. SNPs were then called using a bounded multinomial-based likelihood model ([Catchen *et al.* 2013](#_ENREF_2)). The model accounts for variability in allelic coverage, calculating the likelihood of a locus being heterozygous or homozygous, given the maximum likelihood value of the sequencing error rate (ε); the two alternative genotypes are then tested using a standard likelihood ratio test (*P* = 0.05). To decrease the number of miscalled homozygotes ([Catchen *et al.* 2013](#_ENREF_2)) an upper bound of ε = 0.05 was used, which was deemed suitable based on sequencing error estimated from errors in the barcodes and the control lane of the sequencing run.

Finally, the putative RAD loci found in all 16 individuals were used to create a catalogue of the loci. To account for SNPs fixed within individuals/populations, but variable between them, a mismatch distance of one base was allowed when combining loci across individuals in the catalogue. Each individual was then matched back against the catalogue using SSTACKS to produce a dataset of putative RAD loci and genotypes for each individual at every locus in the catalogue.

A key problem with *de novo* construction of RAD loci is determining the thresholds to use for the clustering parameters. If these parameters are too low, orthologous alleles will not be clustered together within and between individuals, but if the parameters are set too high non-orthologous loci will be incorrectly clustered, resulting in artificial variation if they are fixed for alternative variants ([Catchen *et al.* 2013](#_ENREF_2)). Another issue, arising from sequencing variability between individuals, is determining a minimum locus coverage threshold, which is high enough to reduce error from uncalled heterozygotes, but not so excessive that genotypes from lower coverage individuals are unnecessarily excluded ([Catchen *et al.* 2013](#_ENREF_2)). Therefore, a series of filters were applied to exclude potential paralogues and erroneous genotypes, balanced against retaining sufficient data from all 16 individuals: (i) genotypes supported by less than 10 reads were treated as missing data. (ii) only loci containing a single bi-allelic SNP were retained. (iii) only loci that were genotyped in ≥ 50% of individuals were retained. (iv) loci that were heterozygous in >50% of the individuals genotyped at a given locus were excluded. (v) only loci where the minor allele was genotyped in >1 individual were retained (excluding alleles only found in the single *E. decorata* individual).

From the final filtered set of SNPs a number of datasets were generated allowing for varying degrees of missing data. The aforementioned filtering steps were applied using a combination of commands implemented using the EXPORT_SQL.PL and POPULATIONS modules of STACKS, and additional filtering of the output files produced by these programs using Excel. Data files formatted for subsequent phylogenomic analyses were exported using the POPULATIONS module of STACKS.

After quality filtering 95,874,771 reads were retained from a single lane of sequencing on an Illumina Hi-seq 2000 machine for *de novo* assembly. However, as has been previously reported in other RAD-Seq studies ([Davey *et al.* 2013](#_ENREF_6); [Emerson *et al.* 2010](#_ENREF_8); [Richards *et al.* 2013](#_ENREF_12)), the number of reads retained for each individual was highly variable with an average of 5,992,173 (S.D.=4,123,288) and a range between 15,570,341 and 1,206,901 (Table S1). 13167 biallelic loci were found in eight or more individuals (Table S4), which reduced to 7871 loci once singleton SNPs were removed (or 9781 if singletons of the single *E. decorata* individual are allowed). There were still a substantial number of missing genotypes in this dataset, so to refine the loci used further, only one null was allowed in each of the four main population samples of interest (sinistral and dextral snails from Iwate and Yamagata), leaving 4598 loci. This reduced dataset was used for all subsequent analyses - although not shown here, we found that all other datasets produced similar outputs in terms of subsequent analyses.

**Testing of scenarios via approximate Bayesian computation method.** We used an approximate Bayesian computation (ABC) approach, implemented in the software DIYABC v 2.1.0 ([Cornuet *et al.* 2014](#_ENREF_4)) to compare hypotheses. In brief, simulated data sets were produced for five scenarios, by sampling parameter values in defined prior distributions. Three scenarios were similar in that the population samples show a bifurcating topology, only differing in divergence order. Two other models included ancestral admixture, because the shared chirality between dextral *E. aomoriensis* from Yamagata and Iwate might be because of shared ancestry. The analysis was restricted to the four population samples of *E. quaesita* and *E. aomoriensis*, primarily because the large genetic distance between *E. senckenbergiana* and the other samples meant that it was difficult to find a suitable range of parameter values.

A uniform distribution with large intervals was used for each prior, because we are ignorant of population sizes and divergence times; admixture rate must be relatively low. Specifically, parameters were: populations (10-500000 individuals), t1 (100-1000000 generations), t2 and t3 (10-200000 generations), and rate of admixture (0.001 to 0.10). For each scenario, one million data sets were simulated and the posterior probability estimated by carrying out a regression on 1% of the simulations that were closest to the observed data. The scenario chosen as optimal was that with highest posterior probability. To test whether the optimal model was able to produce data sets similar to the observed one, confidence in the model was assessed by simulating test datasets (pods) and then applying the same procedure to estimate the respective posterior probabilities. Similarly, prior based error analyses were also used to understand the probability with which models might be rejected. Type I and type II errors were assessed by measuring the fraction of data sets simulated under the best scenario that were assigned to other scenarios, and the fraction of data sets simulated under other scenarios that were assigned to the best scenario.

**Mitochondrial phylogenetic and population analyses.** The number of individuals in the phylogenomic analysis was necessarily limited by DNA sequencing resources. To sample more individuals and over a greater geographic area, ~800 bp fragments of 16S rRNA were amplified and sequenced using standard conditions and buffers, including either Takara rTaq or Roche Taq DNA polymerases and primers developed by Chiba([1999](#_ENREF_3)): forward primer: 5’-AAACATACCTTTTGCATAATGG-3’; reverse primer: 5’-CTACGGTCCTTTCGTACTA-3’. Newly generated sequences were edited manually using BIOEDIT version 7.2. All sequences were aligned using T-Coffee ([Notredame *et al.* 2000](#_ENREF_11)), the alignment manually checked, and indels and several difficult to align regions were excluded. The resulting 692 bp sequences were condensed into haplotypes (Table S5, in fasta format and including genbank accessions) using DNASP version 5.1.1 ([Librado and Rozas 2009](#_ENREF_10)). For maximum likelihood phylogenies, an appropriate model of evolution was selected using jModelTest and the Akaike Information Criterion ([Darriba *et al.* 2012](#_ENREF_5)), followed by tree construction and visualisation using PhyML ([Guindon and Gascuel 2003](#_ENREF_9)) and TreeExplorer, including bootstrap support, with the tree rooted on *E. senkenbergiana* ([Davison *et al.* 2005](#_ENREF_7); [Ueshima and Asami 2003](#_ENREF_13)).

Mitochondrial 16S ribosomal RNA sequences were obtained from 329 snails from 76 sites, Combined with the mitochondrial sequence data from two previous studies, specifically 38 samples from 11 sites ([Davison *et al.* 2005](#_ENREF_7)) and 165 samples from 19 sites ([Watanabe and Chiba 2001](#_ENREF_14)), 532 mitochondrial sequences were used from 106 sites across northern and central parts of Japan. From the alignment, 692 bp were retained for phylogenetic and population analyses, condensing into 244 unique haplotypes.

References

Baxter SW, Davey JW, Johnston JS, Shelton AM, Heckel DG, Jiggins CD*, et al.* (2011) Linkage mapping and comparative genomics using next-generation RAD sequencing of a non-model organism. *PLoS One*, **6**, 11.

Catchen J, Hohenlohe PA, Bassham S, Amores A, Cresko WA (2013) Stacks: an analysis tool set for population genomics. *Mol Ecol*, **22**, 3124-3140.

Chiba S (1999) Accelerated evolution of land snails *Mandarina* in the oceanic Bonin Islands: Evidence from mitochondrial DNA sequences. *Evolution*, **53**, 460-471.

Cornuet J-M, Pudlo P, Veyssier J, Dehne-Garcia A, Gautier M, Leblois R*, et al.* (2014) DIYABC v2.0: a software to make approximate Bayesian computation inferences about population history using single nucleotide polymorphism, DNA sequence and microsatellite data. *Bioinformatics*, **30**, 1187-1189.

Darriba D, Taboada GL, Doallo R, Posada D (2012) jModelTest 2: more models, new heuristics and parallel computing. *Nat. Methods*, **9**, 772-772.

Davey JW, Cezard T, Fuentes-Utrilla P, Eland C, Gharbi K, Blaxter ML (2013) Special features of RAD Sequencing data: implications for genotyping. *Mol Ecol*, **22**, 3151–3164.

Davison A, Chiba S, Barton NH, Clarke BC (2005) Speciation and gene flow between snails of opposite chirality. *PLoS Biology*, **3**, e282.

Emerson KJ, Merz CR, Catchen JM, Hohenlohe PA, Cresko WA, Bradshaw WE*, et al.* (2010) Resolving postglacial phylogeography using high-throughput sequencing. *Proc Natl Acad Sci U S A*, **107**, 16196-16200.

Guindon S, Gascuel O (2003) A simple, fast, and accurate algorithm to estimate large phylogenies by maximum likelihood. *Syst Biol*, **52**, 696–704.

Librado P, Rozas J (2009) DnaSP v5: a software for comprehensive analysis of DNA polymorphism data. *Bioinformatics*, **25**, 1451-1452.

Notredame C, Higgins DG, Heringa J (2000) T-Coffee: A novel method for fast and accurate multiple sequence alignment. *J Mol Biol*, **302**, 205-217.

Richards PM, Liu MM, Lowe N, Davey JW, Blaxter ML, Davison A (2013) RAD-Seq derived markers flank the shell colour and banding loci of the *Cepaea nemoralis* supergene. *Mol Ecol*, **22**, 3077-3089.

Ueshima R, Asami T (2003) Single-gene speciation by left-right reversal - A land-snail species of polyphyletic origin results from chirality constraints on mating. *Nature*, **425**, 679-679.

Watanabe Y, Chiba S (2001) High within-population mitochondrial DNA variation due to microvicariance and population mixing in the land snail *Euhadra quaesita* (Pulmonata: Bradybaenidae). *Mol Ecol*, **10**, 2635-2645.
